# A retrospective study of the impact of comorbidity, polypharmacy and demographic factors on patient inclusion and healthcare delivery in phase I oncology trials

**DOI:** 10.1038/s44276-025-00165-y

**Published:** 2025-08-25

**Authors:** Hoda Nemat, Martin Orr, Lucy Barrow, Bindu Raobaikady, Sheila Matharu, Lisa Scerri, Udai Banerji, Ceire Costelloe

**Affiliations:** 1 Health Informatics, Division of Clinical Studies, Institute of Cancer Research, London, UK; 2 Drug Development Unit, The Institute of Cancer Research and The Royal Marsden NHS Foundation Trust, London, UK; 3 Informatics Team, Information Department, The Royal Marsden NHS Foundation Trust, London, UK; 4 Department of Primary Care and Public Health, School of Public Health, Imperial College, London, UK

## Abstract

**Background:**

Phase I trials include patients with metastatic cancer and complex health conditions. Understanding baseline comorbidity and demographic features is critical to improving trial design.

**Methods:**

We used electronic patient records to study the association of comorbidity, polypharmacy, and demographic factors on trial recruitment, time on trial, and health service utilisation.

**Results:**

A cohort of 1671 patients was considered for allocation to a phase I study, of whom 518 patients were recruited to a phase I study and 1153 patients were not. A multivariable analysis revealed polypharmacy was associated with lower recruitment to phase I trials with an odds ratio of 0.95 (95% CI: [0.92, 0.99], *p* = 0.01), and a greater number of emergency admissions with a risk ratio of 1.1 (95% CI: [1.03, 1.17], *p* = 0.01). Interestingly, comorbidity was not associated with lower recruitment but was associated with a lower time on trial with a hazard ratio of 0.75 (95% CI: [0.62, 0.90], *p* ≤ 0.001). Demographic factors, including ethnicity, distance of residence from the hospital, and index of multiple deprivation, did not significantly influence these parameters.

**Conclusion:**

Polypharmacy and comorbidity should be considered both in the design of phase I oncology trials and in planning for healthcare utilisation during these trials.

## Background

Comorbidity refers to the presence of two or more chronic conditions within an individual and poses significant challenges for healthcare systems [1]. In the UK, patients with comorbidity account for most primary care consultations, prescriptions, and hospitalisations [2, 3]. The higher risk of comorbidity in cancer patients results in the risk of polypharmacy and its detrimental impact on their quality of life [4]. Hence, examining polypharmacy along with comorbidity in cancer patients is crucial. Researchers focused on the impact of comorbidity and polypharmacy on the management of patients with a range of cancers and found that either comorbidity, polypharmacy, or both reduced the chance or delayed the institution of systemic anticancer therapy and, in some cases, was correlated to shorter survival [5–7].

Clinical trials are crucial in cancer care. Primarily, to ensure a robust assessment of safety, efficacy, and utility, participants in cancer clinical trials should be representative of the target population. However, comorbidity is prevalent in the population of cancer patients, and it is therefore crucial that cancer patients with comorbidity, who are a complex group with specific health service needs, are considered in trials. Due to the fact that most clinical trials are designed with a single disease in mind, and there is a risk of concealing the potential benefits of experimental treatment, performing such clinical trials has been limited [8–11]. Investigating comorbidity in cancer trial recruitment has rarely been studied. Unger et al. [9] investigated whether clinical trial decision-making and participation were linked to comorbidity in patients with cancer. The study conducted a web-based survey of patients with cancer and assessed multiple recruitment-related outcomes, including discussion of participation, offer of participation, and participation. They found that all the outcomes were negatively impacted by comorbidity.

Phase I trials are conducted primarily to recommend a safe dose/schedule of novel anticancer drugs. These trials are conducted in patients with metastatic cancer who have previously received standard-of-care treatments for their disease and often have significant cancer-related morbidity. Previous studies have explored the impact of sociodemographic factors on recruitment to phase I cancer trials [12, 13]. However, they have not examined the role of comorbidity and polypharmacy in this context. Additionally, the effect of these clinical factors on health service utilisation during trials remains largely unaddressed. We aimed to study if there is evidence that polypharmacy, comorbidity, and sociodemographic features (age, sex, ethnicity, distance to the hospital, and index of multiple deprivation (IMD)) are associated with phase I cancer trial recruitment. Further, we investigated if these factors interact with the experimental treatment to affect other healthcare service use, namely, disease progression through an increase in the discontinuation rate or the number of emergency scans or admissions and length of stay post-admission.

### Methods

#### Study population and data

This retrospective study used electronic patient record (EPR) data from cancer patients referred to the Drug Development Unit (DDU), a joint department at the Royal Marsden NHS Foundation Trust and The Institute of Cancer Research from their local hospitals. All patients seen in the referred and seen in the new patient clinics and subsequently discussed for consideration of recruitment to a phase I trial in the patient allocation meeting (PAM) within 28 days of being seen between 01/10/2018 and 31/12/2021 were considered for analysis. Patients were deemed to have started a phase I study if they had received at least one drug dose. Data on individual patients were collected on the date they were first seen in the new patient clinic and if they were recruited to the phase I trial till the last date they were seen on the trial. If the patient was not allocated or was allocated and found ineligible, data was collected till the last recorded clinic visit in the DDU.

The base dataset was collected from patients on the DDU patient list. It included data from trial enrolment, trial start date, trial end date, death, clinic visit date, age at the time of referral, sex, ethnicity, IMD [14], distance to the Royal Marsden Hospital - Sutton, the number of medications that were not directly used to treat cancer, i.e., excluding chemotherapy, hormonal therapy or immunotherapy, or investigational drug in the clinical trial, the number of diseases, emergency admissions, and emergency scans.

We filtered the dataset based on the closeness of the last clinic visit to the PAM and trial start dates. The threshold to determine the cohort for patients being considered for recruitment was 28 days from the last clinic visit and PAM date. The threshold to determine the trial group was 120 days from the last clinic visit and trial start date. It is worth noting that the threshold for the trial group was larger than that of the recruitment cohort, as we were more concerned with the clinician’s sensitivity to the patient’s baseline characteristics in the decision to recruit into the trial.

#### Data analysis

In our analysis, independent variables comprised sociodemographic features and health-related variables. Sociodemographic features included age at the first clinic appointment in the DDU, sex, ethnicity, IMD score, and distance to the Royal Marsden Hospital - Sutton in miles. Ethnicity was initially recorded in line with the NHS ethnic categories, resulting in small sample sizes for each group. Hence, we recorded this variable as white and non-white groups. Due to non-disclosure, ten missing observations for the ethnicity variable were imputed using the multiple imputation by chained equations algorithm. The IMD was used as a surrogate for income deprivation and had the following components (weighting in brackets): income (22.5%), employment (22.5%), health deprivation and disability (13.5%), education and skills training (13.5%), crime (9.3%), barriers to housing and services (9.3%), and living environment (9.3%). Higher scores for IMD indicate greater deprivation and are assigned from the postcode at registration. Also, distance to the hospital was calculated using patients’ postcodes.

Health-related variables included polypharmacy and comorbidity. There were several options concerning the variable type for polypharmacy and comorbidity. Comorbidity is often quantified using indices such as the Charlson comorbidity index [15] and the Elixhauser comorbidity index [16], primarily developed as prognostic tools. However, both indices apply weighting schemes based on long-term mortality risk, which may not be directly applicable or valid in early-phase oncology trials, where short-term safety and tolerability are the primary endpoints. We chose the number of medications as a surrogate for polypharmacy, which was a simple and reliable variable. Similarly, we initially opted for the number of diseases as a surrogate for comorbidity; however, that resulted in small sample sizes for each group. Hence, comorbidity was treated as a dichotomous variable, indicating the presence or absence of at least one non-cancer condition in individuals with cancer. It is worth noting that in the remainder of this report, we refer to comorbidity as the dichotomous variable.

Both the number of medications and the number of diseases were extracted from free-text patients’ case notes. Disease data was typically in paragraphs, with superfluous data including family medical history and health behaviours. Hence, string manipulation and stop word removal were used to reduce text. The algorithmic extraction of diseases was unfeasible due to synonyms, acronyms, and possible typos. Hence, this was done manually, and the extracted list was validated by clinicians. The extracted diseases were then summed per patient and then grouped into dichotomous for the comorbidity variable. Medications were explicitly listed rather than nested in free text, and punctuation separated terms. There were issues with superfluous text, and the inclusion of complementary medication was deemed unimportant. String manipulation was used to remove additional text, and fuzzy matching was deployed to extract the medications per patient. Medication data was extracted from clinical notes within the patients’ EPR. As such, the dataset may not capture medications prescribed by general practitioners (GPs) or any over-the-counter medications taken by patients. Additionally, it is worth noting that only medicines included in the British National Formulary [17] were extracted.

The outcome variables included recruitment into a clinical trial, time on trial, the number of emergency scans, the number of emergency admissions, and the number of days in the hospital associated with those emergency admissions. The number of emergency admissions and scans collected from patient case notes, and the EPR involved a small set of terms; hence, rules-based algorithms were employed. In patients’ case notes, the data was collected during DDU clinic visits at the patient’s discretion. In the EPR, admissions and scans that were not routine were flagged as emergency. Scans contributed to the number of scans variable, including CT, MRI, PET, X-ray, and ultrasound.

Independent variables were either binary or continuous. Comorbidity, sex, and ethnicity were recorded as binary variables; the number of medications, the number of diseases, age, distance to the Royal Marsden Hospital - Sutton, and IMD score were recorded as continuous variables. The outcome variables were split into binary, continuous, and time-to-event variables. Trial recruitment was coded as a binary variable; the number of emergency admissions, emergency scans, and length of stays after admissions were considered continuous variables, and time on trial was regarded as a time-to-event variable. An event was defined as either having disease progression or death led to a withdrawal.

We aimed to produce statistical models that estimated estimands with minimal bias and consistency. To do so, we needed a directed acyclic graph (DAG) to define the variables required to identify the estimands. Following the guidance from Rodrigues et al. [18] and with the assistance of clinicians, the DAG was defined (supplementary). The DAG included additional observed and unobserved variables to find the adjustment set that fulfilled the backdoor criterion [19]. As validated by dagitty.com [20], the adjustment set involved all independent variables, with additional variables being unadjusted as they were deemed colliders. The DAG was considered general for our outcomes, so the adjustment set was carried over into each statistical model.

#### Statistical analysis

Regression models were used to explore the effect of independent variables on the outcome variables. The analysis was carried out in both univariate and multivariable settings. It is worth noting that in the univariate analysis, the number of diseases and comorbidity were investigated to conduct a comprehensive analysis. However, one of them had to be chosen for the multivariable analysis. Hence, comorbidity was considered for the multivariable analysis. Also, before conducting the primary analysis, we examined whether the number of medications and diseases interacted.

For trial recruitment, as a binary variable, a logistic regression model was used, and odds ratios (ORs) were reported. For the number of emergency admissions, emergency scans, and length of stays after admissions, as continuous variables, Poisson and negative binomial were considered according to the regression-based test for overdispersion [21], and risk ratios (RRs) were reported. Since we found evidence of overdispersion in the number of scans and length of stays, these models were fitted with negative binomial models. Also, the number of emergency admissions variable was fitted with a Poisson model. For time on treatment, as a time-to-event variable, we tested and found evidence for proportional hazards. Hence, a Cox proportional hazards model was fitted, and hazard ratios (HRs) were reported.

## Results

A total of 1671 patients were referred and seen in the new patient clinic of the drug development unit and discussed in the patient allocation meeting for consideration of the phase I trial within 28 days of this initial visit. The patient characteristics are detailed in Table 1. Also, characteristics of outcome variables studied in patients who went on to be recruited to the phase I trial are presented in Table 2.

**Table 1:** Characteristics of all patients in the analysis.

		On trial (*N* = 518)	Not on trial (*N* = 1153)	Overall (*N* = 1671)
**Number of medications**	Mean (SD)	3.4 (3.1)	3.7 (3.2)	3.6 (3.2)
	Median [min, max]	3.0 [0.0, 17.0]	3.0 [0.0, 19.0]	3.0 [0.0, 19.0]
**Number of diseases**	Mean (SD)	1.0 (1.6)	0.8 (1.4)	0.9 (1.5)
	Median [min, max]	0.0 [0.0, 8.0]	0.0 [0.0, 7.0]	0.0 [0.0, 8.0]
**Comorbidity**	Yes	204 (39.4%)	370 (32.1%)	574 (34.4%)
	No	314 (60.6%)	783 (67.9%)	1097 (65.6%)
**Sex**	Male	267 (51.5%)	542 (47.0%)	809 (48.4%)
	Female	251 (48.5%)	611 (53.0%)	862 (51.6%)
**Age**	Mean (SD)	59.7 (12.7)	58.4 (13.3)	58.8 (13.1)
	Median [min, max]	61.0 [18.0, 83.0]	61.0 [18.0, 91.0]	61.0 [18.0, 91.0]
**Ethnicity**	White	441 (85.1%)	948 (82.2%)	1389 (83.1%)
	Non-white	77 (14.9%)	205 (17.8%)	282 (16.9%)
**Distance from the hospital (miles)**	Mean (SD)	68.0 (70.6)	71.6 (66.6)	70.5 (67.8)
	Median [min, max]	45.0 [0.0, 420.8]	49.8 [0.0, 406.5]	48.9 [0.0, 420.8]
**Index for multiple deprivation score**	Mean (SD)	13.8 (10.1)	14.9 (10.0)	14.5 (10.0)
	Median [min, max]	11.1 [0.9, 58.2]	12.3 [0.9, 60.9]	12.0 [0.9, 60.9]

**Table 2:** Characteristics of outcome variables in patients who went on to be recruited to the phase I trial.

	Mean (SD)	Median [min, max]
Time on trial (days)	116.9 (192.2)	56.0 [0.5, 1645.0]
Number of emergency scans	2.8 (5.6)	1.0 [0.0, 50.0]
Number of emergency admissions	0.2 (0.4)	0.0 [0.0, 2.0]
Length of stays for emergency admissions (days)	0.9 (3.8)	0.0 [0.0, 59.0]

Table 1 shows the characteristics of all the patients included in the analysis. It also provides information based on whether the patients started the phase I trial or did not. Also, distributions for the independent and outcome variables are provided in the supplementary material. Moreover, we did not find evidence to support the correlation between the number of medications and diseases, as indicated by Pearson’s and Spearman’s correlation coefficients.

**Fig. 1:**
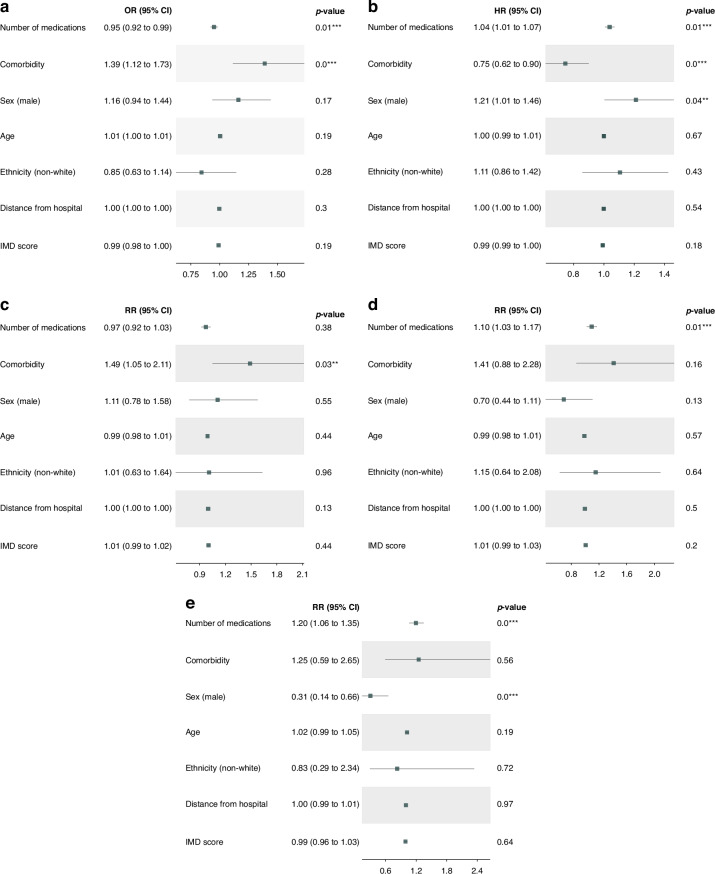
Multivariable analysis of clinical and demographic variables associated with trial-related and health service utilisation outcomes. **a** Variables associated with trial enrolment, **b** variables associated with time on trial, **c** variables associated with number of emergency scans, **d** variables associated with number of admissions, and **e** variables associated with length of stay once admitted.

Fig. 1 shows the results of the multivariable analysis. It is worth noting that the results of univariate analysis are presented in the supplementary. According to Fig. 1a, we identified two variables that were associated with the trial enrolment after adjustment: the number of medications with an OR = 0.954; 95% CI: [0.921, 0.987] and comorbidity with an OR = 1.388; 95% CI: [1.115, 1.728]. The remaining variables showed no association with trial recruitment. In the Cox proportional hazard model for time on trial (Fig. 1b) three variables had statistically significant coefficients: the number of medications with an HR = 1.039; 95% CI: [1.008, 1.071], comorbidity with an HR = 0.747; 95% CI: [0.618, 0.902], and sex (male) with an HR = 1.212; 95% CI: [1.005, 1.461]. In the negative binomial model for the number of emergency scans (Fig. 1c) the presence of comorbidity was associated with an increased risk of additional scans (RR = 1.491; 95% CI: [1.051, 2.115]). In the Poisson model for the number of emergency admissions (Fig. 1d), the greater the number of medications a patient took was associated with an increased risk of emergency admission after adjusting for other covariates (RR = 1.099; 95% CI: [1.028, 1.174]). Also, in the negative binomial model for the length of stays (Fig. 1e), the risk of longer stays post admissions increased with the growth of the number of medications (RR = 1.196; 95% CI: [1.06, 1.349]) but decreased in males (RR = 0.306; 95% CI: [0.143, 0.655]).

### Discussion

To the best of our knowledge, for the first time, we provide the relationship of comorbidity, polypharmacy, and demographic features including ethnicity, distance from the hospital, and IMD score and the outcomes which include patients being recruited to an oncology phase I study and data related to health care utilisation including emergency admissions, emergency scans and time on the clinical trial. This new data will add to published data focusing on baseline organ function measured by blood tests, disease burden measured by imaging, and performance status and outcomes, including response to phase I agents and time on treatment in oncology phase I clinical trials [22–26] to help design and execute oncology phase I trials. Our findings will provide a comparison for future studies evaluating comorbidity, polypharmacy, and demographic factors in the setting of oncology phase I trials.

Our results support the subtle difference between polypharmacy and comorbidity, with our findings indicating that each had distinct relationships with trial recruitment and health service utilisation. Our findings suggest that the higher the number of medications a patient takes, the less likely they will be recruited to phase I clinical trials of an anticancer drug. There could be multiple reasons, for instance, the fact that patients with multiple medications are likely to be in poor health and not meet eligibility criteria for phase I studies. Alternatively, being on various medications could rule patients out of phase I trials of small molecules because of the risk of drug-drug interactions, which are part of the exclusion criteria.

Furthermore, a higher number of medications was associated with a greater risk of emergency admission and a longer length of hospital stay after admission. These arguments align with the fact that patients on multiple medications are in poorer health and survival rates [5]. Interestingly, patients on a larger number of drugs who entered the phase I trial stayed on trial longer. It is possible that the number of medications in our study cohort included multiple medications to control symptoms of cancer, e.g., pain, nausea, vomiting, diarrhoea, infection, and patients who have had many of these symptoms controlled due to a large number of drugs stayed on trials for longer. This needs to be prospectively validated in larger cohorts of patients and may not be relevant outside of cancer trials.

Despite the literature showing a negative effect of comorbidity on cancer trial participation [9], our study surprisingly found that comorbidity was associated with increased odds of being recruited to the phase I cancer trial. Our trial was based on data in a phase I unit, while the published data in this space was based on a web-based survey of patients diagnosed with cancer. The discussion was related to any clinical trial rather than specifically oncology phase I trials, and thus, it may be difficult to compare the two studies. However, it is encouraging to observe that comorbidity was not associated with a lower likelihood of enrolment into oncology phase I trials. This finding aligns with ongoing calls to reduce overly restrictive eligibility criteria in clinical trials, which often exclude patients with comorbidity. Broadening inclusion criteria could help ensure that trial populations more accurately reflect ‘real-world’ patients, rather than a select group of ‘fit’ individuals, thereby improving the generalisability of trial results and maximising benefits to patients in society when drugs are licensed [27, 28]. Moreover, this result may reflect a distinction between referral and enrolment processes. While comorbidity may pose a barrier to referral in broader cancer trials, it may not necessarily hinder enrolment once patients are referred to phase I oncology trials. It is, however, possible that referring clinicians make assumptions of not referring patients to oncology clinical trials if they have chronic infections like HIV/Hep C or chronic heart failure. However, in our study, comorbidity was associated with reduced time on a trial and increased need for emergency scans. This implies that cancer patients with comorbidity are less likely to benefit from trials requiring increased healthcare resources.

On examination of patient characteristics, we found that males in our dataset had more time on trial and a much lower risk of longer stays after admissions. Despite evidence in the literature suggesting that social deprivation and ethnicity have been associated with poor recruitment to clinical trials and reduced inclusivity [29–31], our study found no such associations for sociodemographic factors, including age, ethnicity, and IMD score. Moreover, distance to the hospital, which is often presumed to be a barrier to recruitment, did not affect patients’ odds of recruitment to phase I cancer trial, remaining on trial, or utilising healthcare resources, according to our multivariable analysis. It is important to clarify that the findings of this study indicate no evidence of selection into trials based on the specified characteristics once patients are referred. However, the study did not investigate referral patterns.

It is worth noting that our study population is skewed towards less deprived areas, as indicated by a higher proportion of participants residing in areas with lower levels of deprivation based on IMD rankings, compared to the national distribution. This may limit the generalisability of our findings to more deprived populations, and discrepancies may emerge in larger datasets encompassing broader geographical areas. From the registration data, the ethnicity of the population studied showed a population of 83% white, which is slightly greater than the national average of 81% [32]. However, because of the relatively small numbers of patients from ethnic minorities, it is difficult to know if such patients have a lower recruitment to phase I trials from this analysis. Further studies in areas with a more ethnically and socioeconomically diverse population can be benchmarked against this study.

The outcomes of this study could be improved if we had access to drugs prescribed by GPs. While electronic patient records of patients being considered for phase I studies are very detailed due to regulatory reporting required for such studies, it will be key to have access to GP records in larger population-based studies as hospital electronic patient data may not be as detailed as for patients on phase I clinical trials. This study was conducted in a tertiary referral oncology hospital and a specialised phase I unit. While many of the findings could be potentially generalisable to oncology-specific phase I practice, however, given the differences in requirements of frequent follow up, infrastructure to monitor toxicity (workforce specialisation and healthcare professional-to-patient ratio), our findings may not be generalisable to randomised phase III oncology trials, which are typically focused on efficacy, and conducted in secondary or tertiary care centres.

Phase I studies in oncology are conducted in patients with advanced cancer who have complex health needs. Building on existing research into sociodemographic factors influencing recruitment to phase I oncology trials, this study provides new insights into the role of comorbidity, polypharmacy, and sociodemographic factors in both trial recruitment and subsequent healthcare utilisation. These findings can inform the design of phase I oncology trials by guiding eligibility criteria and helping to plan the healthcare resources needed to support trial participation in patients with cancer.

## Supplementary information

Supplementary material

## Data Availability

The data supporting this study's findings was from the drug development unit at the Royal Marsden Hospital NHS Foundation Trust. The study data was stored within the secure collaborative workspace in the Royal Marsden’s Trusted Research Environment, BRIDgE. Data are available on reasonable request and compliance with both individual hospital trusts and the UK Health Research Authority conditions as well as individual academic institutions data access approval processes.

## Code availability

The analysis was conducted using Python 3.6. The data supporting this study’s findings was from the drug development unit at the Royal Marsden Hospital NHS Foundation Trust. The study data was stored within the secure collaborative workspace in the Royal Marsden’s Trusted Research Environment, BRIDgE. The code and data used in this study are not publicly available.
