# Supplementary material for: A retrospective study of the impact of comorbidity, polypharmacy and demographic factors on patient inclusion and healthcare delivery in phase I oncology trials
Source: BJC Rep. 2025 Aug 25;3:57. doi: 10.1038/s44276-025-00165-y (PMC12378352; doi:10.1038/s44276-025-00165-y)
Supplement: Supplementary file 1 — Supplementary material [file 44276_2025_165_MOESM1_ESM.docx]

Supplementary material

Supplementary Figure 1: Distributions of independent variables

(A) number of medications, (B) number of diseases, (C) sex, (D) age, (E) ethnicity, (F) distance from hospital, and (G) IMD score.

| 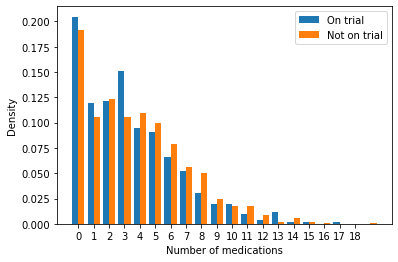 | 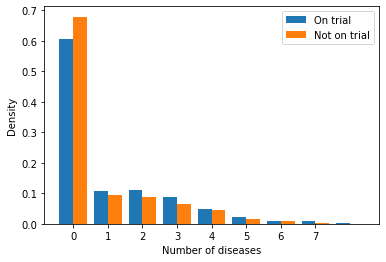 |
| --- | --- |
| (A) | (B) |
| **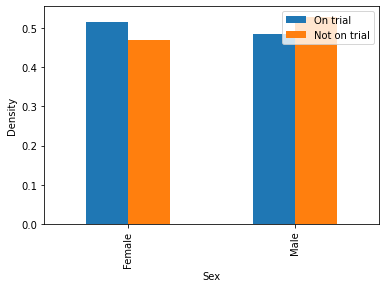** | **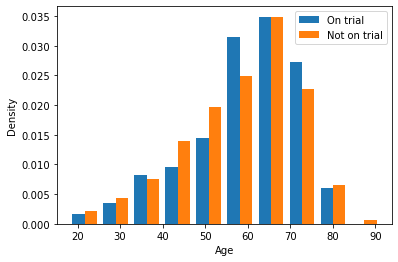** |
| (C) | (D) |
| **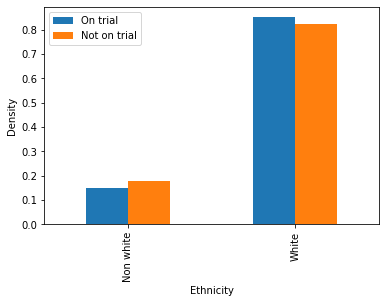** | **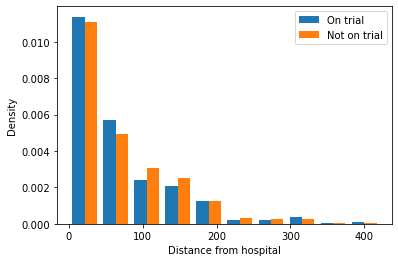** |
| (E) | (F) |
| **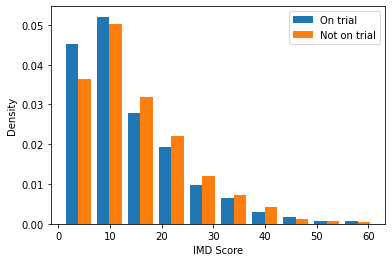** | |
| (G) | |

Supplementary Figure 2: Distribution of outcome variables

(A) number of emergency scans, (B) number of admissions, and (C) length of stay after admissions.

| 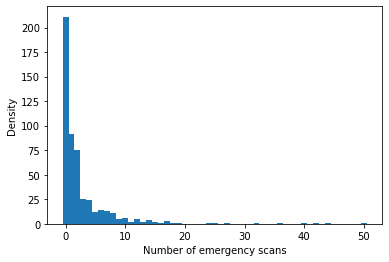 | 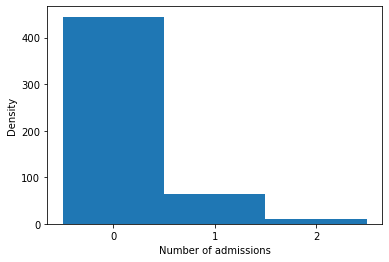 |
| --- | --- |
| (A) | (B) |
| 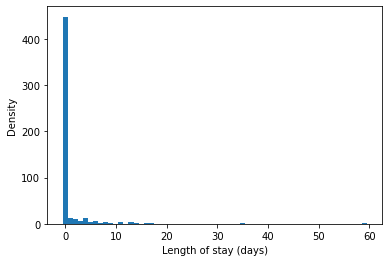 | |
| (C) | |

Supplementary Figure 3: The Kaplan Meier graph of time on trial


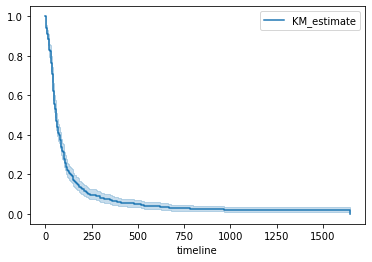


Supplementary Figure 4: Directed acyclic graph with both observed and unobserved variables


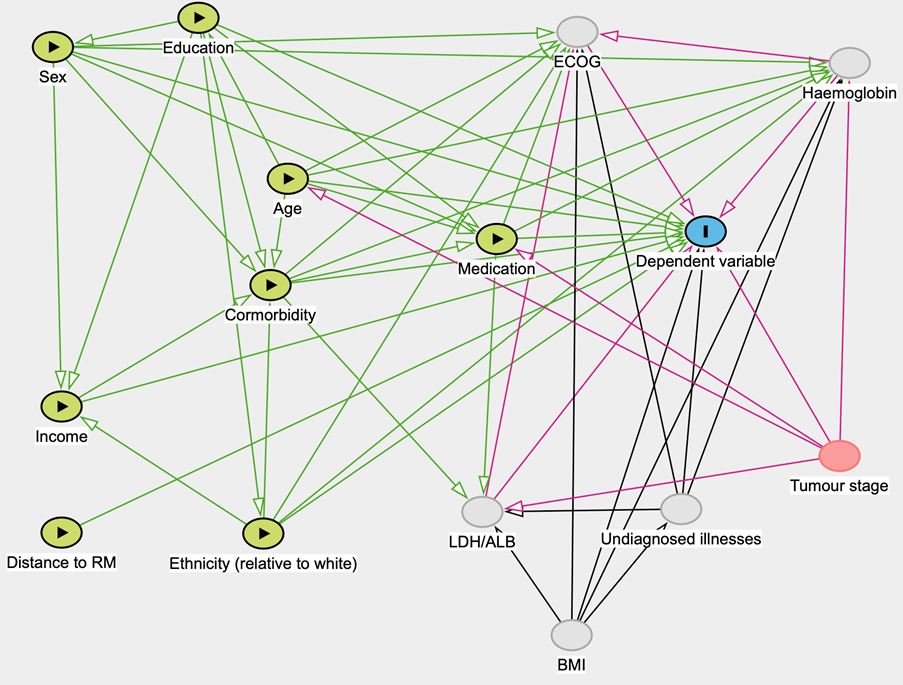


Supplementary Figure 5: Results of univariate analysis

A) variables associated with trial enrolment, B) variables associated with time on trial, C) variables associated with number of emergency scans, D) variables associated with number of admissions, and E) variables associated with length of stay once admitted.

| 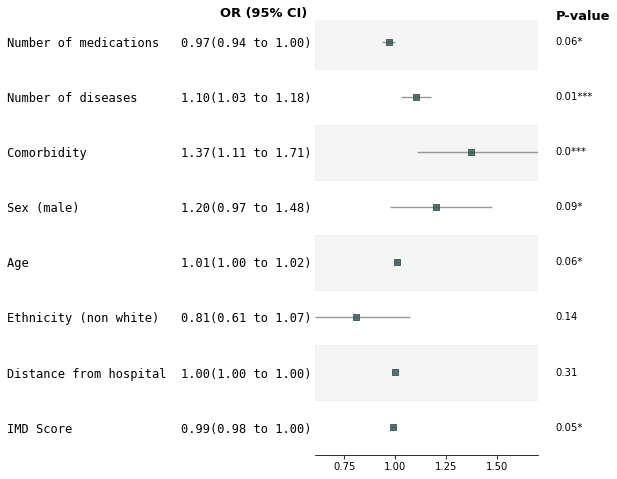 | 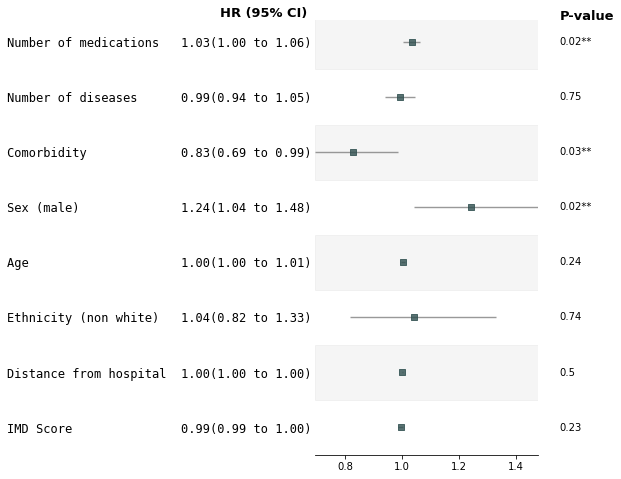 |
| --- | --- |
| (A) | (B) |
| 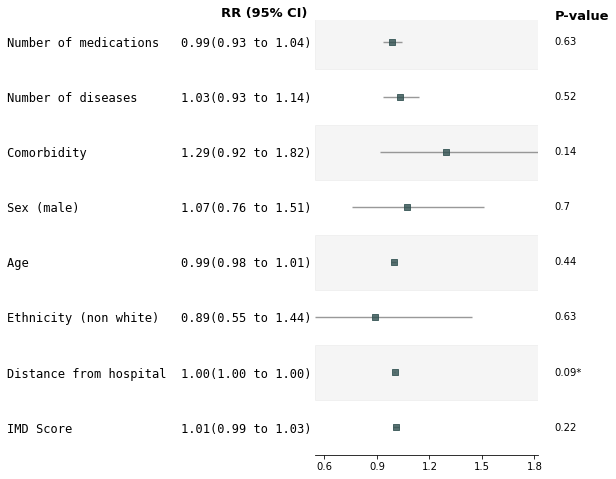 | 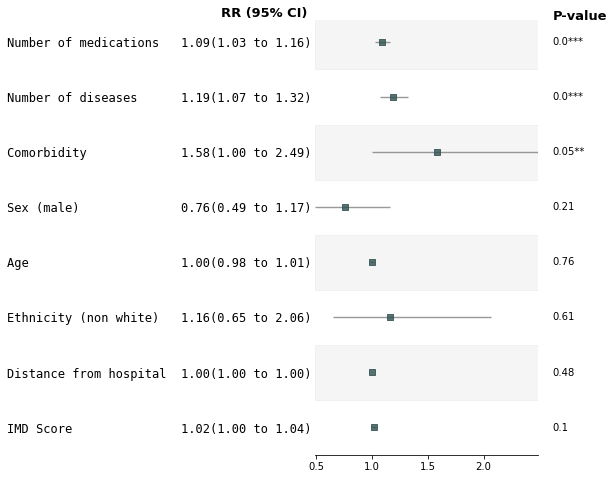 |
| (C) | (D) |
| 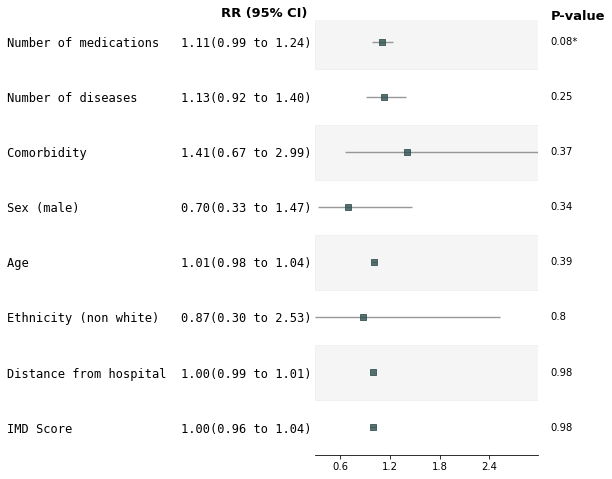 | |
| (E) | |
